# Comparison of prognosis between neoadjuvant imatinib and upfront surgery for GIST: A systematic review and meta-analysis

**DOI:** 10.3389/fphar.2022.966486

**Published:** 2022-08-29

**Authors:** Zhen Liu, Zimu Zhang, Juan Sun, Jie Li, Ziyang Zeng, Mingwei Ma, Xin Ye, Fan Feng, Weiming Kang

**Affiliations:** ^1^ Department of General Surgery, Peking Union Medical College Hospital, Chinese Academy of Medical Sciences and Peking Union Medical College, Beijing, China; ^2^ Division of Digestive Surgery, Xijing Hospital of Digestive Diseases, Air Force Medical University, Xi’an, China

**Keywords:** gastrointestinal stromal tumor, neoadjuvant imatinib, upfront surgery, R0, prognosis, meta-analysis

## Abstract

**Background:** Significant survival benefit of adjuvant imatinib therapy has been observed in gastrointestinal stromal tumor (GIST). However, the impact of neoadjuvant imatinib on prognosis of GIST remains unclear. This meta-analysis aimed to compare the prognostic impact between upfront surgery and neoadjuvant imatinib plus surgery on GIST.

**Methods:** A comprehensive literature search was performed to identify eligible studies up to 30 Sep 2021, through PubMed, Embase, Web of Science, and Cochrane Library. Studies compared the impact of upfront surgery and neoadjuvant imatinib plus surgery on disease-free (DFS) or overall survival (OS) in patients with GIST were selected.

**Results:** Seven eligible studies with 17,171 patients were included. The reduction rates of tumor size in rectal and mixed site GIST were 33% and 29.8%, respectively. Neoadjuvant imatinib was not significantly associated with DFS compared with no-neoadjuvant therapy in rectal GIST (HR: 0.71, 95% CI: 0.35–1.41). The OS of rectal GIST was significantly improved by neoadjuvant imatinib compared with no-neoadjuvant therapy (HR: 0.36, 95% CI: 0.17–0.75).

**Conclusion:** Neoadjuvant imatinib therapy contributed to tumor shrinkage and R0 resection of rectal GIST. Neoadjuvant imatinib plus surgery significantly improved overall survival of rectal GIST in comparison with upfront surgery.

## Introduction

Gastrointestinal stromal tumor is one of the most common mesenchymal tumors arising from the gastrointestinal tract (GI), with an annual incidence of 10 cases per million people globally which accounts for 1–3% of cancers in the entire GI (1, 2). GIST is considered to develop from the gain-of-function mutations of KIT ((Hirota et al., 1998)) and platelet-derived growth factor receptor alpha (PDGFRA) (Heinrich et al., 2003) and can occur anywhere of the GI. The most common site is stomach (60–70%), followed by small intestine (20–30%) and colorectum (5%) (Rubin et al., 2007; Joensuu et al., 2013; Liu et al., 2018).

Surgical resection remains the first choice of curative treatment for primary GIST. Since the first report (Joensuu et al., 2001) of the use of imatinib for metastatic GIST in 2001, various tyrosine kinase inhibitors have been growingly developed and used in clinical treatment of GIST ((Demetri et al., 2002; Demetri et al., 2006; Demetri et al., 2013; Blay et al., 2020; Chen et al., 2020; Heinrich et al., 2020; Chen et al., 2021a)). During this period, significant survival benefit has been observed in those with high-risk GIST who received adjuvant imatinib after surgery (Joensuu et al., 2020). These positive results brought attention to the use of neoadjuvant imatinib for GIST with large size or in special anatomic site. Neoadjuvant therapy has been demonstrated to contribute to the improvement of survival of several malignancies (Das, 2017; Cai et al., 2018; Mittendorf et al., 2020; Chen et al., 2021b). Till now, several retrospective and single-arm studies have reported the feasibility and effectiveness of neoadjuvant therapy on GIST ((Wang et al., 2012; Ling et al., 2021a; Renberg et al., 2022; Wong et al., 2022)). Recent guidelines recommended consideration of neoadjuvant imatinib therapy for patients if the surgical morbidity could be reduced preoperatively (Casali et al., 2018; von Mehren et al., 2020). However, the impact of neoadjuvant imatinib on prognosis of GIST remains unclear due to the absence of strong evidence from randomized controlled trials. Thus, the current meta-analysis aimed to review the relevant literature and provide a comprehensive view of the survival influence of neoadjuvant imatinib on GIST.

## Material and methods

### Search strategy

A systematic search of literature using keywords as “gastrointestinal stromal tumor,” “GIST,” “neoadjuvant,” “preoperative treatment” and “preoperative therapy,” was carried out by two investigators (ZL and ZZ) through PubMed, Embase, Web of Science and Cochrane Library to identify studies that compared the treatment effect between neoadjuvant therapy and upfront surgery for GIST. The search was updated to 30 Sep 2021. Attempts have been made to get additional eligible studies through searching the references of relevant studies. This study was in compliance with the Preferred Reporting Items for Systematic Reviews and Meta-analyses (PRISMA) guideline (Liberati et al., 2009).

### Selection criteria

Eligible studies were identified by two investigators (ZL and JS) according to the following criteria: (Miettinen et al., 2003) Participants (P): Patients were diagnosed pathologically and immunohistochemistrically as primary GISTs; (Connolly et al., 2003); Interventions (I) and comparisons (C): Patients received neoadjuvant imatinib followed by surgery and/or adjuvant therapy in research group and upfront surgery and/or adjuvant therapy in control group. The outcomes were compared between research and control groups; (Heinrich et al., 2003); Outcomes (O): Disease-free survival (DFS) and/or overall survival (OS) were/was available or able to be calculated by sufficient data in the studies. When duplicate studies from same center were identified, only the newest or largest study was included. Any discrepancies were resolved by discussion with a third investigator (ZYZ).

### Data extraction

The first author, publication year, country, sample size, tumor site, information of neoadjuvant imatinib, surgery, resection margin, adjuvant therapy, follow-up, DFS and OS were extracted independently by two investigators (SWOY and JL). If the hazard ratio (HR) and 95% confidence interval (CI) were not provided in the studies, we either emailed the corresponding author for original results or calculated these data from the Kaplan-Meier survival curves using the methods reported by Tierney et al. (Tierney et al., 2007). A third observer (MWM) engaged in discussions to resolve any controversial issues.

### Quality assessment

Two authors (ZL and ZZ) independently assessed the quality of all included studies using the Newcastle-Ottawa Quality Assessment Scale (NOS) (Stang, 2010) with the highest score of nine, and any discrepancies in the scores were resolved by discussion with a third reviewer (JS).

### Statistical analysis

The pooled survival data were measured using the HR and 95% CI. Some HRs and 95% CIs were extracted from Kaplan-Meier curves using Engauge Digitizer (version 4.1). Statistical heterogeneity was evaluated using the chi-square test and I^2^ statistics. Subgroup analysis was conducted to identify the source of heterogeneity. The random-effects model was used by default because of the nature of these retrospective studies. The estimated results of the fixed-effects model are also provided for reference. Sensitivity analysis was performed to validate the stability of the model by sequentially omitting each study. The publication bias was not performed as fewer than ten studies were included. Statistical analyses were performed using R software 3.6.1 (R Project for Statistical Computing) with the meta package (4.13-0) (Balduzzi et al., 2019). A two-sided *p* < 0.05 was considered significant. The GRADE profiler software (version 3.6) was used to estimate the level of evidence (Guyatt et al., 2008).

## Results

### Eligible studies in the meta-analysis

**FIGURE 1:**
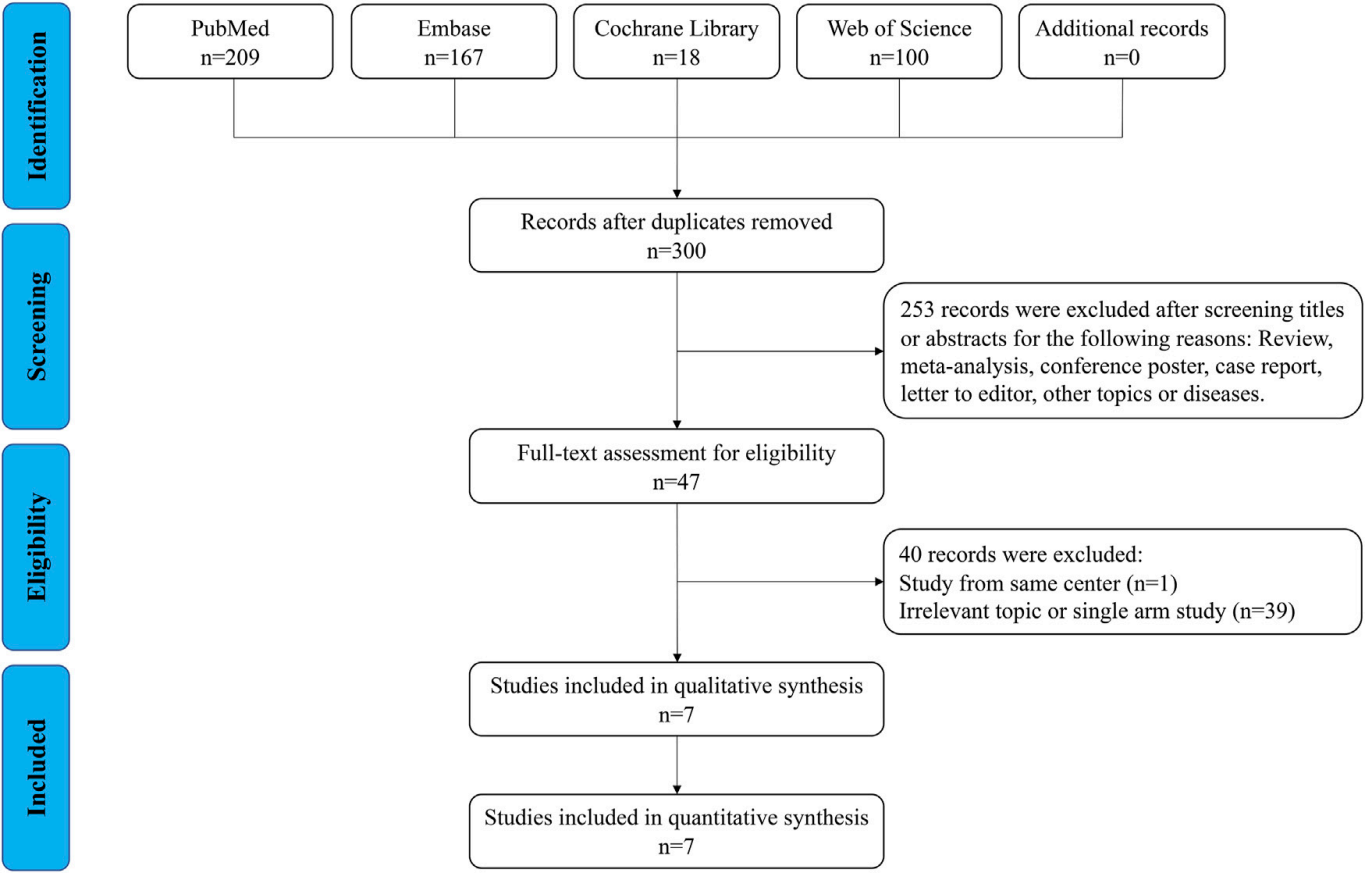
Flow chart of the search strategy.

As shown in Figure 1, 494 relevant publications were identified through the literature search. After screening and assessment, seven eligible studies (Hawkins et al., 2017; Yan et al., 2018; NS et al., 2020; Yang et al., 2020; Ling et al., 2021b; Marqueen et al., 2021; Yang et al., 2021) with 17,171 patients were included in this meta-analysis (Table 1 and Supplementary Table S1). There were 1178 patients who received neoadjuvant therapy, and 15,993 patients who received upfront surgery. None of these patients experienced preoperative metastasis. Patients in both groups received adjuvant therapy accordingly. Two studies reported their median reduction rate of tumor size were 29.8% (mixed sites) and 33% (rectum) (Supplementary Table S1), respectively. And in study of Yang 2021 (38), the median tumor size of rectal GIST reduced from 5.8 to 3.8 cm after the use of neoadjuvant imatinib. The NOS scores of the studies ranged from seven to eight, indicating their relatively high quality of methodology. The GRADE evidence profiles of three indicators (resection margin, DFS and OS) were presented in Supplementary Table S2.

**TABLE 1:** Summarization of the seven included studies.

Study	Country	Site	Metastasis	Sample size	Neoadjuvant imatinib	Upfront surgery	Follow-up (medain, mo)	NOS
Hawkins 2016								7
Rectum-NCDB	USA	Rectum	No	74	21	53	NA	
Yan 2018	China	Mixed^a^	No	191	47	144	NA	8
Ijzerman 2020	Netherlands	Rectum	No	109	78	31	28 (0–115)	8
Yang 2020	China	Rectum	No	64	29	35	41 (1–122)	8
Ling 2021	China	Rectum	No	85	52	33	36.8 (12.7–152.7)	8
Marqueen 2021								8
Total-NCDB	USA	Mixed^b^	No	16,308	865	15,443	44.5 (IQR 22.1–72.5)	
Stomach-NCDB		Stomach	No	10,635	583	10,052		
Yang 2021	China	Rectum		340	86	254	49 (6–215)	8

NCDB, National Cancer Database; NOS, Newcastle–Ottawa Quality Assessment Scale.

a Mixted: stomach, intestine and enterocoelia.

b Mixed: stomach, esophagus, small bowel and colorectum.

### Resection margin

**FIGURE 2:**
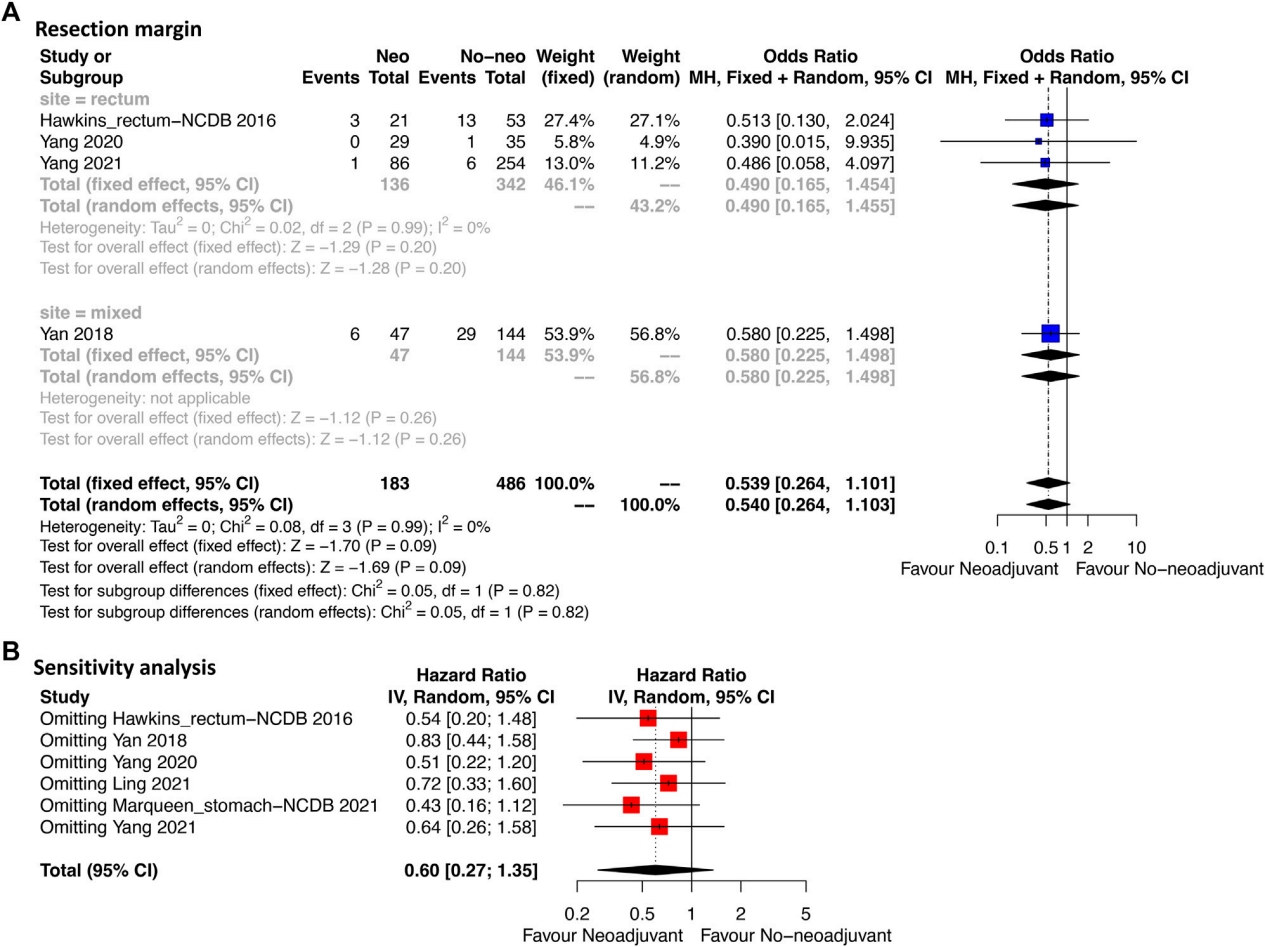
Forest plots illustrating resection margin between neoadjuvant and no-neoadjuvant imatinib **(A)** and sensitivity analysis **(B)**.

Four studies provided the information of margin resection. Among them, three studies analyzed rectal GIST and one analyzed mixed site GIST (stomach, intestine and enterocoelia). Figure 2A revealed that the R0 resection rate had no significant difference between neoadjuvant imatinib and no-neoadjuvant therapy (HR: 0.54, 95% CI: 0.26–1.10; *p* = 0.99, I^2^ = 0%; reference: no-neoadjuvant therapy). In the subgroup analysis of rectal GIST, a trend of higher R0 resection rate ranging from 85.3% to 98.8% was observed in neoadjuvant imatinib group compared with the rate ranging from 74.4% to 92.0% in no-neoadjuvant therapy group (Supplementary Table S1). But the difference was not statistically significant (HR: 0.49, 95% CI: 0.17–1.46; *p* = 0.99, I^2^ = 0%; reference: no-neoadjuvant therapy; Figure 2A). Sensitivity analysis was performed by omitting each study sequentially, and the estimated results did not differ significantly, indicating the stability of the model (Figure 2B).

### Disease-free survival

**FIGURE 3:**
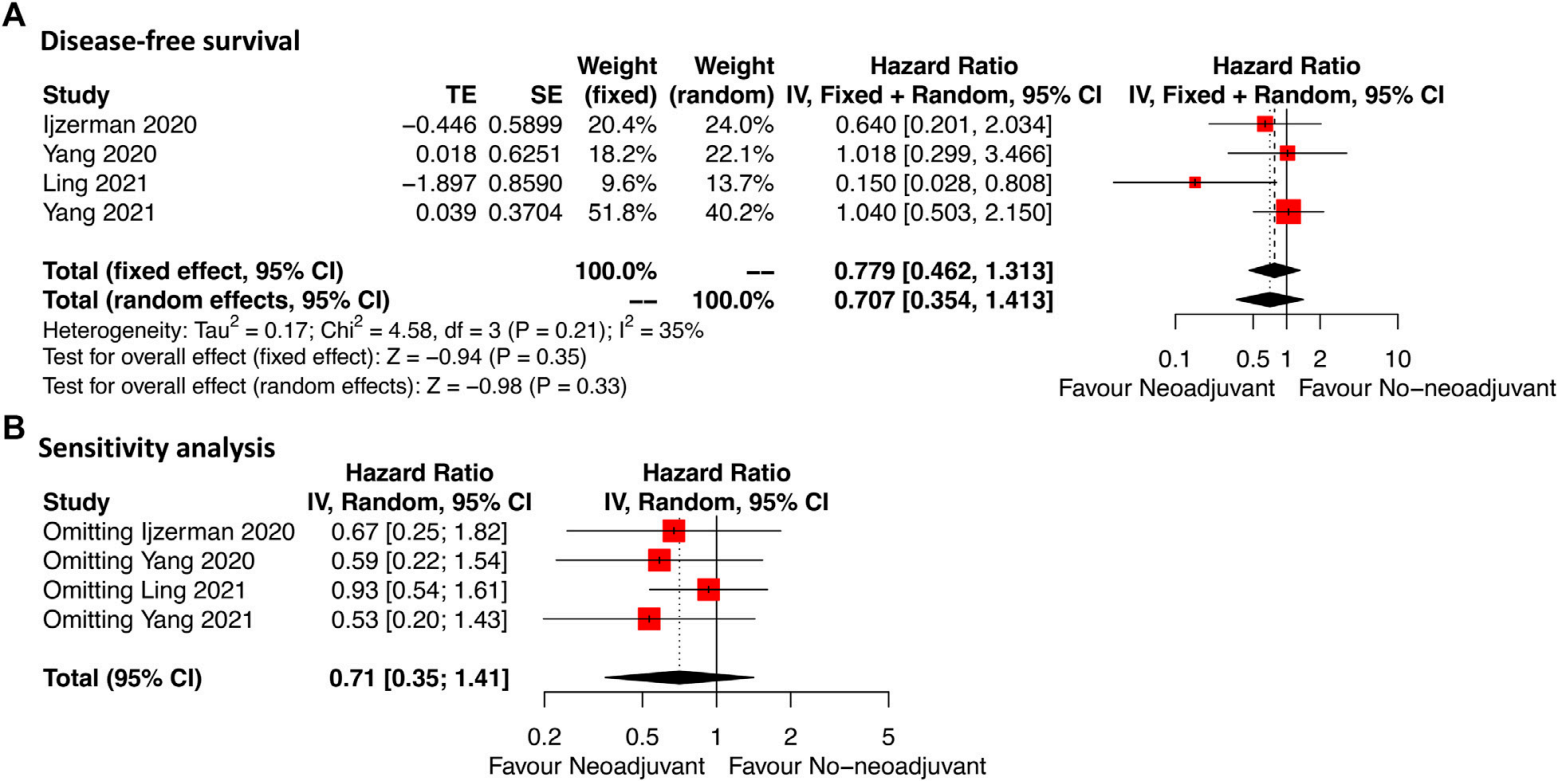
Forest plots illustrating disease-free survival between neoadjuvant and no-neoadjuvant imatinib **(A)** and sensitivity analysis **(B)**.

As shown in Figure 3A, DFS data were available in four studies of which the included cases were all rectal GIST. Neoadjuvant imatinib was not significantly associated with DFS compared with no-neoadjuvant therapy (HR: 0.71, 95% CI: 0.35–1.41; reference: no-neoadjuvant therapy), which was consistent with the estimated results of the fixed-effects model (HR: 0.78, 95% CI: 0.46–1.31; reference: no-neoadjuvant therapy), indicating the absence of heterogeneity among studies (*p* = 0.21, I^2^ = 35%). Sensitivity analysis was performed by omitting each study sequentially, and the estimated results did not differ significantly, indicating the stability of the model (Figure 3B).

### Overall survival

**FIGURE 4:**
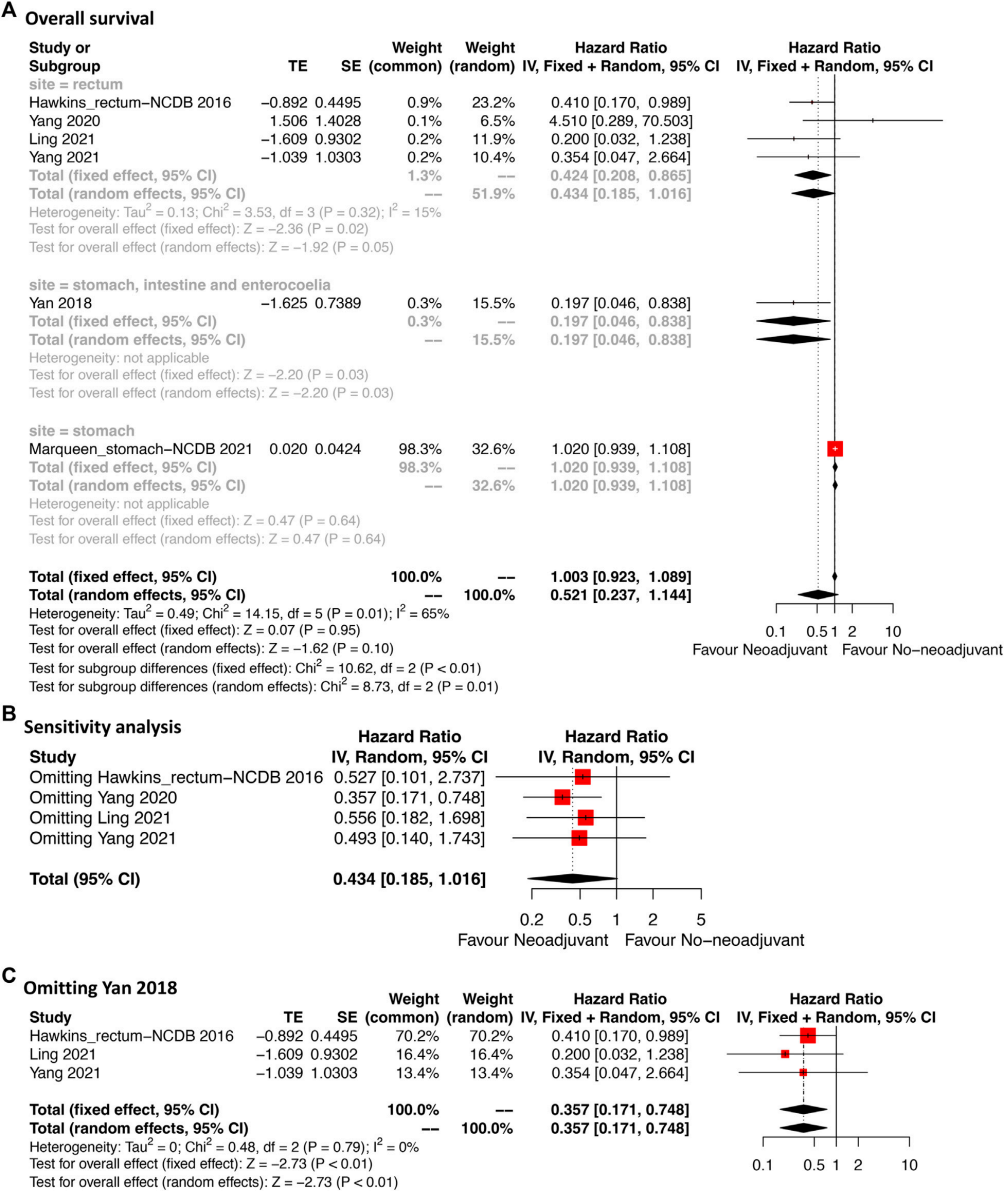
Forest plots illustrating overall survival between neoadjuvant and no-neoadjuvant imatinib **(A)** and sensitivity analyses **(B**,**C)**.

Six studies providing OS data were included (Figure 4A). For the total cases, patients who received neoadjuvant imatinib had similar OS compared with those who received no-neoadjuvant therapy (HR: 0.52, 95% CI: 0.24–1.14; reference: no-neoadjuvant therapy). However, a moderate heterogeneity was observed (*p* = 0.01, I^2^ = 65%). To identify the potential source of heterogeneity, subgroup analysis was performed according to tumor site. A significant decrease of heterogeneity was observed in the subgroup of rectal GIST (*p* = 0.32, I^2^ = 15%). In this subgroup, neoadjuvant imatinib was significantly associated with better OS compared with no-neoadjuvant therapy (HR: 0.43, 95% CI: 0.19–1.02), which was consistent with the estimated results of the fixed-effects model (HR: 0.43, 95% CI: 0.21–0.87). Neoadjuvant imatinib significantly improved OS in mixed site GIST (HR: 0.20, 95% CI: 0.05–0.84) but not in gastric GIST (HR: 1.02, 95% CI: 0.94–1.11).

Sensitivity analysis was performed by omitting each study sequentially in rectal GIST subgroup. The result after omitting Yang 2020 was significantly different from that after omitting other three studies which might weaken the credibility of the model (Figure 4B). This might due to the different including criteria in Yang et al.’s study (Yang et al., 2020). Their study mainly compared the transanal and nontransanal surgery for rectal GIST. The prognostic value of neoadjuvant therapy was only analyzed in the multivariate cox model. After omitting the study of Yang 2020 (Figure 4C), the effected result was stable (HR: 0.36, 95% CI: 0.17–0.75) and the heterogeneity additionally decreased (*p* = 0.79, I^2^ = 0%) indicating the credibility of the result.

## Discussion

The present study compared the clinical effect of neoadjuvant imatinib and upfront surgery on GIST. The benefits of tumor shrinkage as well as improvement of R0 resection rate in rectal GIST were observed after the use of neoadjuvant imatinib therapy. Neoadjuvant imatinib was not significantly associated with DFS compared with no-neoadjuvant therapy in rectal GIST. However, neoadjuvant imatinib significantly improved OS of rectal GIST compared with no-neoadjuvant therapy.

The use of neoadjuvant imatinib has been reported to yield benefits in downstaging to avoid extensive resection in cases of bulky tumors or tumors in particular site, such as rectum (Andtbacka et al., 2007; Fiore et al., 2009; Nishida et al., 2019). A previous observational study (Wilkinson et al., 2015) reported that tumor size and mitotic index significantly reduced after receipt of neoadjuvant imatinib in rectal GIST which allows for less extensive sphincter-preserving surgery. Several studies reported that the sphincter-preserving rate in rectal GIST was 33.3–100% after treatment of neoadjuvant therapy (Kaneko et al., 2019). In the phase II APOLLON trial (Hohenberger et al., 2012), 64% of patients received a less radical surgery after 6 months treatment of neoadjuvant imatinib. In current meta-analysis, two studies reported their median reduction rate of tumor size were 29.8% (mixed sites) and 33% (rectum) (Supplementary Table S1), respectively. And in study of Yang 2021 (38), the median tumor size of rectal GIST reduced from 5.8 to 3.8 cm after neoadjuvant imatinib. The shrinkage of tumor size is considered to contribute to the achievement of R0 resection.

Complete resection is one of the primary concerns in the treatment of GIST ((Schmieder et al., 2016)). The R0 resection rate was previously reported to be 77.3–100% after treatment of neoadjuvant therapy (Kaneko et al., 2019). In the phase II RTOG 0132 study (Wang et al., 2012) including 31 cases of primary GISTs, a 68% rate of R0 resection (21 cases) was reported within the median neoadjuvant therapy duration of 9.9 weeks. Kurokawa et al. (Kurokawa et al., 2017) reported another phase II study with an achievement of 90% R0 resection rate in large gastric GIST treated with neoadjuvant therapy. This high rate of R0 resection was attributed to the long neoadjuvant therapy duration of 6 months. Several studies suggested the best duration of neoadjuvant therapy for maximal tumor response is 6–12 months (Bonvalot et al., 2006; Haller et al., 2007; Kurokawa et al., 2017). Which is in line with the 6 months duration or more recommended by NCCN guidelines (von Mehren et al., 2020).

In current meta-analysis, four studies reported the duration of neoadjuvant imatinib which ranged from 6.3 to 10 months (median) indicating a relatively optimal window for tumor response (Supplementary Table S1). The partial response rate in rectal GIST was reported to be 65.9% and 75% by Ling 2021 (36) and Yang 2021 (38), respectively. What is more, the disease control rate achieved 100% in Ling’s study (Ling et al., 2021b). Yang et al. (Yang et al., 2021) further reported that the effect of neoadjuvant imatinib is dependent on the genetic type and KIT exon 11 mutation responds better than other types which suggested the importance of genetic sequencing. In the rectal GIST subgroup of current study, a trend of higher R0 resection rate ranging from 85.3% to 98.8% was observed in neoadjuvant imatinib group compared with that ranging from 74.4% to 92.0% in no-neoadjuvant therapy group, though the difference was not significant.

Prognosis is another main indication in the evaluation of efficacy of neoadjuvant therapy which has not been sufficiently reported previously. Hawkins et al. (Hawkins et al., 2017) analyzed 333 cases of rectal GIST enrolled in NCDB, and the multivariate analysis showed that neoadjuvant therapy was not related with OS. But in the subgroup of tumors that were larger than 5 cm and received radical resection, neoadjuvant therapy had a significantly higher 5-year OS than no-neoadjuvant therapy (79.2% vs. 51.2%). Ling et al. (Ling et al., 2021b) also demonstrated that neoadjuvant therapy not only reduced tumor size of rectal GIST, but also improved 5-year distant recurrence-free survival and disease-specific survival. But the information of resection margin was not available in their study. In the contrary, a recent multicenter research (Yang et al., 2021) including 340 cases of rectal GISTs from 11 centers in China reported that, the 3-year rates of DFS and OS of those who received neoadjuvant therapy were 95% and 100%, respectively, which were similar in comparison with those of patients who received no-neoadjuvant therapy. Future updated follow-up is warranted for this multicenter study.

In current meta-analysis, DFS was available in four studies which were all focusing on rectal GIST. The pooled results showed that DFS was not significantly associated with neoadjuvant imatinib in rectal GIST. It is reported that positive margin is possibly associated with the recurrence of GIST but this negative impact disappeared in the era of imatinib due to the use of adjuvant imatinib (Liu et al., 2022). This comparable DFS between neoadjuvant imatinib group and no-neoadjuvant therapy group in current meta-analysis might partly due to the balanced rate of R0 resection between the two groups and the proper use of adjuvant imatinib in both groups. However, the OS was significantly improved after receipt of neoadjuvant imatinib in patients with rectal GIST in present study.

Limitations existed in current study. Firstly, due to the retrospective nature of these eligible studies, some inherent bias in the study design and process cannot be avoided. Secondly, tumor size and mitotic index as well as their reduction rate after neoadjuvant imatinib were not provided in all eligible studies that an overview of prognostic factors in GIST other than neoadjuvant imatinib was not available. Thirdly, detailed information of tumor rupture and adjuvant therapy were not able to be analyzed which were key factors impacting the prognosis of GIST. Fourthly, five out of the seven studies analyzed rectal GIST so further studies focusing on GISTs in other sites are warranted. Fifthly, a multicenter study including 340 cases of rectal GIST from 11 centers in China were included in this meta-analysis which provided relatively firm results for decision-making of neoadjuvant imatinib therapy. However, randomized controlled trials are still lacking and warranted to clarify the role of neoadjuvant imatinib in treatment of GIST.

## Conclusion

Rectal GIST benefits from neoadjuvant imatinib regarding to the achievements of tumor shrinkage and R0 resection. Although neoadjuvant imatinib had no significant advantage on the disease-free survival, patients with rectal GIST who received neoadjuvant imatinib plus surgery had better overall survival than those who received upfront surgery.

## Data Availability

The original contributions presented in the study are included in the article/Supplementary Material, further inquiries can be directed to the corresponding author.

## Abbreviations

DFS, disease-free survival; GIST, gastrointestinal stromal tumor; GRADE, the Grading of Recommendations Assessment, Development, and Evaluation system; NCCN, National Comprehensive Cancer Network; NOS, Newcastle-Ottawa Quality Assessment Scale; OS, overall survival; R0, microscopically negative resection margin.
