# Supplementary material for: Comparison of prognosis between neoadjuvant imatinib and upfront surgery for GIST: A systematic review and meta-analysis
Source: Front Pharmacol. 2022 Aug 29;13:966486. doi: 10.3389/fphar.2022.966486 (PMC9465640; doi:10.3389/fphar.2022.966486)
Supplement: Supplementary file 1 [file DataSheet1.docx]

Table 1 Summarization of the treatments and responds.

| Study | Site | Neoadjuvant  imatinib | Upfront  surgery | Tumor size  (median, cm) | Distance to anus  (median, cm) | Duration of  Neoadjuvant  imatinib  (median, mo) | Tumor  response | Reduction  Rate of size  (median, %) | Adjuvant  therapy | R0 resection rate |
| --- | --- | --- | --- | --- | --- | --- | --- | --- | --- | --- |
| Hawkins 2016 |  |  |  |  |  |  |  |  |  |  |
| Rectum-NCDB | Rectum | 21 | 53 | >5cm | NA | NA | NA | NA | Yes | Neo: 85.3%  Surg: 74.4% |
| Yan 2018 | Mixed* | 47 | 144 | >3cm | NA | NA | NA | 29.8 (1.5-52.2) | Yes | Neo: 87.2%  Surg:79.9% |
| Ijzerman 2020 | Rectum | 78 | 31 | 6.1 (1.4-25) | 3.5 (0-8) | 10 (1-102) | NA | 33 (20-100) | Yes | 62% |
| Yang 2020 | Rectum | 29 | 35 | 38 cases 2-5cm  18 cases >5cm | TA: 4.2±0.9  NTA: 5.8±2.1 | NA | NA | NA | Yes | Neo: 100%  Surg: 97.1% |
| Ling 2021 | Rectum | 52 | 33 | Neo: 36 cases >5cm Surg: 25 cases >5cm | Neo: 19 cases >3cm  Surg: 16 cases >3cm | 6.9 (1-58.9) | DCR: 100%  PR: 65.9%  SD: 34.1% | NA | Yes | NA |
| Marqueen 2021 |  |  |  |  |  |  |  |  |  |  |
| Total-NCDB | Mixed** | 865 | 15443 | Neo: 691 cases >5cm Surg: 7521 cases >5cm | NA | 6.3 (3-NA) | NA | NA | Yes | Neo: 89.0%  Surg: 92.0% |
| Stomach-NCDB | Stomach | 583 | 10052 | NA | NA | NA | NA | NA | Yes | NA |
| Yang 2021 | Rectum | 86 | 254 | 5 (1.7-12.6) | 4 (91% low rectum) | 9 | PR: 75%  SD: 18% | PR: 5.8 to 3.8cm | Yes | Neo: 98.8%  Surg: 97.6% |

NCDB: National Cancer Database; NOS: Newcastle–Ottawa Quality Assessment Scale; Neo: Neoadjuvant imatinib; Surg: Upfront surgery; TA: Transanal surgery; NTA: nontransanal surgery; DCR: Disease control rate; PR: Partial response; SD: Stable disease; NA: Not available.

*Mixted: stomach, intestine and enterocoelia.

**Mixed: stomach, esophagus, small bowel and colorectum.

Table 2 GRADE profile evidence.

| Quality assessment | | | | | | |  | № of patients | |  | Effect | Quality | Importance |
| --- | --- | --- | --- | --- | --- | --- | --- | --- | --- | --- | --- | --- | --- |
| № of studies | Study design | Risk of bias | Inconsistency | Indirectness | Imprecision | Publication bias |  | Neo | No-neo |  | Relative (95% CI) |  |  |
| Margin4 | observational studies | not serious | not serious | not serious | not serious | NA |  | 951 | 14928 |  | 0.97 (0.48-1.97) | ⨁⨁⨁⊝ moderate | critical |
| DFS  4 | observational studies | not serious | not serious | not serious | not serious | NA |  | 245 | 353 |  | 0.71 (0.35-1.41) | ⨁⨁⊝⊝ low | important |
| OS  6 | observational studies | not serious | not serious | not serious | not serious | NA |  | 818 | 10571 |  | 0.52 (0.24-1.14) | ⨁⨁⨁⊝ moderate | critical |

DFS: Disease-free survival; OS: Overall survival; NA: Not available; Neo: Neoadjuvant therapy; No-neo: No-neoadjuvant therapy with upfront surgery; CI: Confidence interval.
